# An Intracranial Extradural Dermoid Cyst Presenting with Two Dermal Sinuses and an Abscess in a Child

**DOI:** 10.1155/2021/9917673

**Published:** 2021-07-26

**Authors:** Aysha Albastaki, Reem AlThawadi, Janan Alajaimi, Khawla F. Ali, Talal Almayman

**Affiliations:** ^1^Royal College of Surgeons in Ireland-Medical University of Bahrain, Busaiteen, Bahrain; ^2^King Hamad University Hospital, Al Sayh, Bahrain

## Abstract

Dermoid cysts account for only a small fraction of intracranial masses, with extradural dermoid cysts being considered a much rarer entity than those located intradurally. Intracranial dermoid cysts vary in clinical presentations: some maybe asymptomatic whilst others harbor features of raised intracranial pressure, neurological deficits, or even aseptic meningitis. Dermoid cysts may also present with cutaneous lesions. Herein, we report a rare case of a 1-year-old female presenting with a midline, scalp abscess. Brain MRI revealed an intracranial, extradural tumor, with features suggestive of a dermoid cyst, unusually located in the crista galli, and complicated by the formation of two cutaneous sinus tracts. After identification and characterization by MRI, bitemporal craniotomy was performed with complete excision of the mass and sinus tracts. Histological analysis confirmed dermoid cyst as the final diagnosis. Postoperatively, the patient recovered fully and had no evidence of recurrence in subsequent visits. The case mentioned above highlights the rarity of such a presentation for an intracranial extradural dermoid cyst and the vitality of early imaging for midline cutaneous lesions for identification of intracranial extensions and avoidance of detrimental consequences.

## 1. Introduction

Dermoid cysts are benign lesions lined by the squamous epithelium that congenitally arise as a result of failure of the ectoderm to separate from the neural tube. These cysts might also form subsequent to trauma that led to the sequestration and inclusion of ectodermal tissue [1].

Embryologically, dermoid cysts are derived from two germ cell layers: the ectoderm and the mesoderm. Dermoid cysts typically appear along the midline plane of the human body, corresponding to sites of embryonic fusion [2].

Intracranial dermoid cysts are relatively rare, benign tumors, which make up 0.025% to 0.04% of all intracranial tumors, and are seen in 1 in 2500 live births [3]. Two-thirds of all intracranial cysts arise in the posterior fossa, with the remaining third usually located intradurally in the parasellar and midline frontobasal region [2, 4]. Extradural dermoid cysts, on the other hand, are considered a much rarer entity [5, 6].

Herein, we report a rare case of an extradural, intracranial dermoid cyst, located on the crista galli, presenting with a scalp abscess and complicated by the formation of two dermal sinuses. We review and discuss in details the current literature in view of this rarity.

## 2. Case Presentation

A 1-year-old female presented to the Department of Pediatric Surgery with a progressively enlarging forehead swelling for the past 3 weeks. On examination, the swelling was erythematous, tender, fluctuant, 3 × 4 cm in diameter, and located to the right of the midline, consistent with the diagnosis of an abscess. Concurrently, a smaller lesion on the nasal bridge containing a few hair strands was present. The nasal lesion, per the mother's report, had been present since birth.

**Figure 1:**
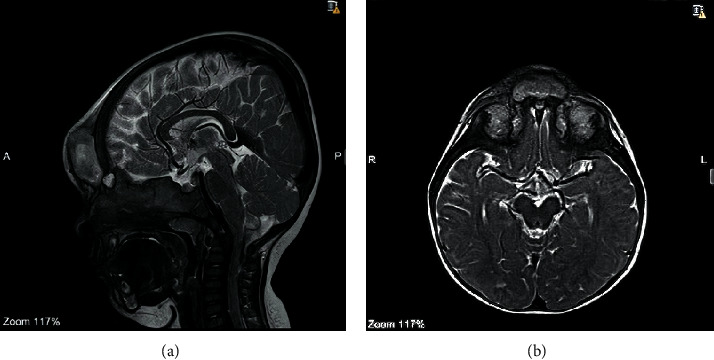
The patient's brain MRI in the T2-weighted sequence. The intracranial mass connected to the sinus tract and opening into the forehead abscess are shown in a (a) sagittal plane and (b) transverse plane.

A brain MRI was also performed to rule out any possible intracranial extension of the lesion, given its midline location. The MRI revealed an intracranial, extradural tumor, with radiological features suggestive of a dermoid cyst. Additionally, the lesion was complicated by the formation of two dermal sinus tracts, with one opening into the forehead and the other into the nasal region (Figure 1).

Subsequently, the patient underwent surgical drainage, with a small incision made at the medial aspect of the right eyebrow, revealing copious amounts of pus.

**Figure 2:**
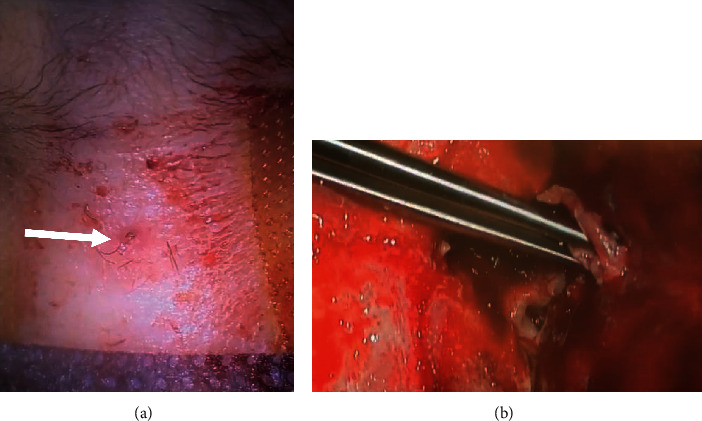
Gross appearance of the lesion. (a) The cutaneous lesion with a few hair strands on the nasal bridge (white arrow). (b) Intraoperative image showing the sinus tract that was leading to the forehead, being held by the surgical forceps.

The decision to surgically excise the intracranial mass and debride both the frontal and nasal dermal sinuses was agreed upon by both neurosurgery and ENT departments. Intraoperatively, a sutar bitemporal incision of the scalp was performed, followed by bitemporal craniotomy, exposing the dura mater. The frontal mass, overlying the crista galli, was identified with one tract that led to the forehead and another extending to the nasal lesion (Figure 2). Because the mass was fused with the parietal dura, extra care was taken to precisely excise it using a sharp surface cut in order to avoid cerebrospinal fluid (CSF) leakage. Although the mass was fused with the outermost layer of the dura, dural invasion was highly unlikely as ruled out by both radiological evidence and the absence of any symptoms in the patient suggestive of chemical meningitis. The mass was successfully excised, intact with no rupture or CSF leakage, and the dermal sinus that led to the forehead was removed. The lower nasal sinus tract was excised by ENT, and the overlying skin was sutured. Lastly, a Hemovac drain was applied and the bone flap was reconstructed. The subcutaneous layer of the scalp skin was approximated with absorbable sutures, and the overlying skin was closed with proline interrupted sutures.

Histopathology reports on the specimen obtained intraoperatively confirmed the diagnosis of a dermoid cyst with a chronically inflamed dermal sinus tract. Postoperatively, the patient had full recovery with no complications. During subsequent follow-up visits to the neurosurgery clinic, there were no clinical features suggestive of possible recurrence.

## 3. Discussion

### 3.1. Background

Intracranial dermoid and epidermoid cysts have not been acknowledged until E. Bostroem first recognized in 1897 the epithelial origin of the “tumeur perlees” that was described by Cruveilhier in 1829 [7, 8]. Ever since these discoveries, several reports of dermoid and epidermoid cysts have been published [9].

Intracranial dermoids and epidermoids are rare benign congenital tumors compromising 0.4% to 1.9% in several large series of intracranial tumors [10, 11]. Dermoids, however, appear less frequently than epidermoids with a ratio of 1 : 1.2–1.9 and account for only 0.025% to 0.04% to of all intracranial tumors [3, 12].

Location of head and neck dermoid cysts, both extradural and intradural, has been mainly reported in the fontanels, petrous apex, Eustachian tube, periorbital or perinasal soft tissue, cavernous sinus, anterior cranial fossa, or in the skull suture located near the skin where they connect directly to a dermal sinus [5, 6, 13, 14]. Head and neck dermoid cysts rarely originate from the skull base, the infratemporal region, supratentorial, or the fourth ventricle [15–18].

Extradural intracranial dermoid cysts, when compared to those located intradurally, are a much rarer entity [5, 6]. Only one case was reported of a dermoid cyst located in the vicinity of the foramen ovale [13]. To our knowledge, this is the first report of a dermoid cyst lying on the crista galli with two dermal sinuses.

### 3.2. The Origins of Dermoid Cysts

The embryologic origins of dermoid cysts have been controversial due to the unknown mechanism of formation of such dysraphisms. Dermoid cysts are made up of both ectodermal and mesodermal elements and form either as a result of incomplete closure of the neural tube during the third to fifth week of fetal development or due to traumatic implantation of skin elements [13, 15, 19].

As the fetus develops, anomalous closure of the neural folds in the midline results in an aberrant adhesion between the ectoderm and the underlying neural tube. This incomplete separation forms a cyst dwelling of a well-differentiated stratified squamous epithelium, hair follicles, apocrine, sebaceous and sweat gland, smooth and stratified muscle, cartilage, bone, minor salivary glands, nerves, and lymph nodes [4, 19, 20].

The provenance of intracranial dermoid cyst cells may be explained by two main theories. Firstly, it has been proposed that dermoid cysts might originate from multipotent embryonic stem cells. The other theory, however, states that the cysts might arise from the translocation of epithelial cells that have migrated from the otic vesicles or developing neurovasculature. Although these stem cells were not found in the dura, they can still form an extradural cyst by proliferation in combination with epithelial cell migration during the extradural neurovasculature process [4].

### 3.3. Clinical Presentation of Dermoid Cysts

The average time between the onset of symptoms and diagnosis of dermoid cysts has been reported at 8.5 years within the previous century, allowing copious time for a variety of clinical symptoms to appear [21, 22]. However, with the recent advances in diagnostic imaging, the diagnosis of dermoid cysts has become possible in its preclinical stages and as early as infancy [13, 23, 24].

Given that intracranial dermoid cysts are space-occupying lesions, their clinical presentation depends on the size and location of the tumor. Symptoms, if present, are often nonspecific and may include headaches, dizziness, or seizures. Papilledema is unlikely to occur, and the patients' spinal tap is clear in more than half of the cases [10, 25]. Patients with dermoid cysts might also be asymptomatic with the only clinical sign being a cutaneous lesion, as was the case presented in this report. This, hence, highlights the importance radiological imaging at determining the presence of any intracranial extension to midline lesions.

Despite their benign nature, dermoid cysts have a high rate of morbidity and mortality if rupture was to occur [26]. Rupture, whether spontaneous or traumatic, results in the lipid content of the cyst, a chemical irritant, to be released into the subarachnoid space and ventricles [27–29]. Following rupture, patients would present with severe aseptic or chemical meningitis leading to transient cerebral ischemia secondary to vasospasm and eventually leading to infarction, coma, and death [30–33]. The rupture of the cyst might also lead to chronic granulated arachnoiditis, aqueductal stenosis, or ventriculitis [23, 34]. However, with the early radiological detection of dermoid cysts, devastating events such as the latter are rarely reported.

Other complications of dermoid cysts include the development of dermal sinuses, which are channels connecting the dermoid cyst with the skin. Lesions as such most commonly present in the lumbar and occipital regions [35]. Intracranial extension of the nasal dermal sinuses is a rare entity, with the first case being reported in 1951 by Matson and Ingraham [36].

The differential diagnosis of dermoid cysts includes a wide range of pathologies, such as epidermoid cysts, lipomas, teratomas, cystic craniopharyngiomas, and arachnoid cysts [26, 32, 37]. The differentiation between these multiple lesions can be achieved by the aid of radiological imaging. However, for definitive diagnosis, histological examination of the lesion is necessary.

### 3.4. Diagnosis of Dermoid Cysts

Both CT and MRI scans are considered to be the diagnostic methods of choice for dermoid cysts. The radiological features associated with dermoid cysts include the characteristic signal intensities, absence of perilesional oedema, and the presence of well-defined margins to the cyst.

CT scans can easily depict dermoid cysts as discrete lesions of low density, a characteristic which is consistent with their fatty content. Mass effects are usually minimal relative to the size of the lesions. In addition, peripheral calcifications may be spotted in the lucent area [12]. The wall of the cyst can partially enhance after administering CT-iodinated contrast material [32]. However, enhancement is generally uncommon and if present, usually only exists as a thin peripheral rim [38]. However, on rare occasions, dermoid cysts may appear as hyperdense lesions on a CT scan [39, 40]. This unusual feature of hyperdensity is thought to be due to a combination of microcalcification, blood products, and saponification, and this is more likely to occur in the posterior fossa, for reasons which are unknown [38].

On MRI scans, dermoid cysts have variable signal characteristics [38]. They tend to appear as hyperintense lesions on T1-weighted imaging due to their lipid content and as a heterogeneous lesion on T2-weighted imaging owing to their various contents that include bones, cartilage, and calcifications [26, 32, 33]. Hence, as aforementioned, the signal intensity on MRI images can be influenced by the variety of contents present within the dermoid cysts [13, 41, 42].

Other imaging modalities, such as plain skull film, may show a well-defined, lucent lesion with ring-like or egg shell calcification, as well as bony defects, if present.

### 3.5. Management of Dermoid Cysts

Complete surgical resection of dermoid cysts is the only effective treatment for prevention of future recurrence and/or complications [30]. Resection has to be carefully performed to avoid spilling of contents intracranially. In some instances, complete resection might be difficult due to the cyst's extensive fibrous adhesions into the adjacent neurovascular structures [13]. Despite this, total resection of the cyst along with the adhesions and sinuses is crucial as to avoid recurrences.

## 4. Conclusions

In summary, we reported a rare case of an extradural, intracranial dermoid cyst, located on the crista galli, presenting with a scalp abscess and complicated by the formation of two dermal sinuses. The case highlights the rarity of such a presentation. It also emphasizes the necessity of early radiological imaging in the evaluation of midline, scalp lesions for determination of the presence or absence of intracranial extension. The latter constitutes a critical prevention method against future catastrophic intracranial events.

## Data Availability

No data were used to support this study.
